# Impact of a Small Cell on the RF-EMF Exposure in a Train

**DOI:** 10.3390/ijerph120302639

**Published:** 2015-02-27

**Authors:** Sam Aerts, David Plets, Arno Thielens, Luc Martens, Wout Joseph

**Affiliations:** Department of Information Technology, Ghent University/iMinds, Gaston Crommenlaan 8 box 201, B-9050 Ghent, Belgium; E-Mails: david.plets@intec.ugent.be (D.P.); arno.thielens@intec.ugent.be (A.T.); luc.martens@intec.ugent.be (L.M.); wout.joseph@intec.ugent.be (W.J.)

**Keywords:** radio frequency electromagnetic fields, RF-EMF exposure assessment, base station, small cell, measurement, dose, train, emerging technology

## Abstract

The deployment of a miniature mobile-phone base station or small cell in a train car significantly improves the coverage and the capacity of a mobile network service on the train. However, the impact of the small cell on the passengers’ exposure to radio-frequency electromagnetic fields (RF-EMF) is unknown. In this study, we assessed experimentally the RF-EMF exposure of a mobile-phone user who is either connected to the outdoor macrocell network or to an in-train small cell, while traveling on the train, by means of the absorbed-dose concept, which combines the base station downlink exposure with the mobile-phone uplink exposure. For Global System for Mobile Communications (GSM) technology at 1800 MHz, we found that by connecting to a small cell, the brain exposure of the user could realistically be reduced by a factor 35 and the whole-body exposure by a factor 11.

## 1. Introduction

In 2012, no less than 220 million people travelled by train in Belgium, a 40% increase in the number of person kilometers in a single decade [1]. Parallel to this significant growth in public transport, there has also been a huge upsurge in mobile telephony and data traffic. However, in our society that is becoming increasingly dependent on wireless communication, the railway environment is lagging behind; most commonly noticed by the fact that during a train ride maintaining a steady mobile connection (voice or data) still regularly proves to be problematic [2]. Meanwhile, several studies on the exposure of the general public to radio-frequency (RF) electromagnetic fields (EMF) have established that public transport (bus, train, *etc.*) has become the dominant micro-environment in terms of RF-EMF exposure, with the largest RF-EMF strengths attributed to mobile-phone use [3,4,5,6].

In essence, both of these issues are caused by the same factors; *i.e.*, (1) the fast movement of the train, forcing the mobile phone to repeatedly connect to a different base station (macrocell) (*i.e.*, a handover), during which the power of the mobile device is set to its maximum [7]; (2) the metal frame of the train that behaves more or less like a Faraday cage, significantly attenuating any signal that penetrates it (hence, any mobile device inside the train is forced to radiate stronger for the transmitted signal to possess enough power to reach the base station); and (3) the large amount of people present in a small environment which is the train car, increasing the chances of mobile-phone use, and thus reinforcing the aforementioned factors.

A combined solution that would effectively eliminate the first two factors is to bring the mobile-phone base station inside the train. This can be done by deploying a small cell in the train car, a miniature Wi-Fi-like base station (with a maximum output power of about 100 mW) to which mobile-phone users can connect directly and continuously, instead of repeatedly connecting to far-off macrocell base stations.

However, although previous studies in static indoor environments have shown a potentially reduced whole-body exposure by installing a small cell [8,9], the actual effect of the small cell on the RF-EMF exposure of train passengers remains uncertain, while the general public might already feel hesitant about the presence of a base station in the train car [10]. In this study, we aimed at quantifying the small cell’s impact on the RF-EMF exposure, in the body and in the brain, of a mobile-phone user in a moving train, by combining the subject’s exposure to the mobile phone and to the base station(s).

## 2. Methods

### 2.1. A Mobile-Phone User on A Moving Train—Exposure Scenarios

**Figure 1:**
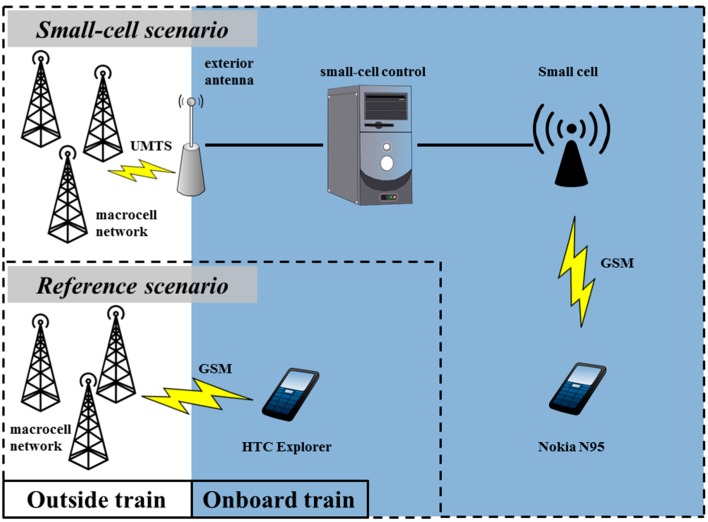
Schematic of the connection setups on the moving train: the reference scenario consisted of an HTC Explorer connected to the Proximus GSM macrocell network; the small-cell scenario consisted of a Nokia N95 connected to an in-train GSM small cell, which was connected to the Proximus UMTS macrocell network through an exterior roof antenna.

Our assessment of the impact of a small cell on the human RF-EMF exposure in a train consisted in comparing the exposure of a single mobile-phone user during two scenarios: (a) the *reference scenario*, in which the subject’s mobile phone is connected to a static macrocell network outside the train; and (b) the *small-cell scenario*, in which the mobile phone is connected to a small cell deployed in the subject’s train car. See Figure 1 for a schematic overview of the two scenarios.

In this study, we considered the following sources of RF-EMF: (1) the subject’s mobile phone (a near-field source); (2) the base station to which the mobile phone was connected (a far-field source), and in case of the small-cell scenario; (3) the macrocell as well, as it is still present and radiating. We did not consider, however, other macrocell base stations, mobile phones of other persons, or the small-cell-to-macrocell-connection antenna on the roof of the train (Figure 1). The implications of these omissions are discussed further on in Section 3.3.

The *small-cell scenario* consisted of a Huawei (Shenzhen, Guangdong, China) BTS3900B small cell, placed on the luggage rack inside a train car (1.8 m above the floor) and attached to an exterior roof antenna, which connected it to the Proximus (Brussels, Belgium) UMTS macrocell network (Figure 1). The output power of the miniature base station (*P_SC_*) was fixed at 50 mW (17 dBm) (the maximum *P_SC_* of this small cell is 200 mW), and it transmitted solely in the Global System for Mobile Communications technology at 1800 MHz (GSM1800) band, more specifically at a downlink frequency of 1808.4 MHz (data provided by Proximus). Furthermore, the subject’s mobile phone was of the “Nokia N95” (Espoo, Finland) type, on which an application called *Field Test Display* was installed, enabling us to monitor the mobile phone’s received and transmit powers [8]. Connected via the small cell, the mobile phone transmitted at a corresponding uplink frequency of 1713.4 MHz.

The *reference scenario* (Figure 1) involved a mobile phone of the type “HTC Explorer” (HTC Corporation, Taoyuan City, Taiwan), connected to the Proximus network. As we focused on GSM technology in this study, the mobile phone was set to “Only GSM” mode, which ensured that it would only connect via the GSM900 (GSM at 900 MHz) or GSM1800 bands. The HTC Explorer was equipped with the application *Azenqos* (developed by Freewill FX Company Limited, Bangkok, Thailand), which enabled us to log various mobile-connection parameters

The study was performed in a single-deck train car of the type “MS96”, built by Bombardier (Montreal, Canada) and Alstom (Levallois-Perret, France), and put at our disposal by the NMBS (“Nationale Maatschappij der Belgische Spoorwegen”, Brussels, Belgium). The train was in service on the Ghent–Eupen track (a route of approximately 200 km) in Belgium, moving at an average speed of ~85 km/h.

### 2.2. Exposure Assessment

In order to compare the subject’s total exposure during the two scenarios of Figure 1, it was essential to combine the base stations’ *downlink* and the mobile phone’s *uplink* exposure into a single exposure proxy. Hence, we used the framework presented by Lauer *et al.* [11], which has been successfully applied in [8,12] to study the influence of a small cell on the exposure in an office environment, and consists in calculating the RF-EMF dose *D* (in J/kg) absorbed by the subject during the considered scenarios.

#### 2.2.1. Power Measurements

To calculate the subject’s absorbed dose (see Section 2.2.2), accurate estimates of the power received (from base stations) as well as the power transmitted by a mobile phone during the train ride were required. To this end, we used the monitoring applications *Field Test Display* on the mobile phone in the small-cell scenario, and *Azenqos* on the mobile phone in the reference scenario.

Given the large variations anticipated in both received and transmit power of a mobile phone along any train trajectory when connected to an outside macrocell network, the reference scenario measurements were conducted *continuously* throughout the train ride, so as to obtain the power distributions along the trajectory. For this purpose, 159 voice calls were established to the “Speaking Clock” (a recorded voice service that gives the correct time), lasting on average 68.6 s (with a standard deviation of 5 s), while the *Azenqos* application continuously logged the following parameters: the frequency band of the connection, the Received Signal Strength Indication or *RSSI* (in dBm), and the transmit or uplink power, *P_UL_* (in dBm).

The small-cell scenario measurements, on the other hand, were performed only once, as the indoor environment did not vary throughout the train ride. During a voice call, the *RSSI* and *P_UL_* were measured at nine positions along the corridor of the train car (all in line-of-sight of the small cell), between 1 and 14 m from the small cell. With the mobile phone held horizontally by the experimenter on the palm of his hand (at approximately 1.3 m above the floor and 0.3 m from the body, with the upper arm held to the body and the lower arm at a 90 degrees angle), *RSSI* and *P_UL_* were captured along four orthogonal orientations at each measurement position [8], after which the median of the linear power values at the four orientations was retained as measurement value at the respective position to account for the influence of the mobile antenna directivity [8,13].

It is important to note that *RSSI* is merely a measure of the power present in the received signal, and that there is no direct link known beforehand between the measured *RSSI* value at a certain position and the actual received power, *P_R_*, at this position. In this study, we calibrated our *RSSI* measurements to the correct *P_R_* using the train car path loss model for mobile-network frequencies of Aerts *et al.* [14]. Additionally, the measured transmit power values *P_UL_* (in W) were divided by 8 to account for the Time Division Multiple Access (TDMA) nature of GSM communications (*i.e.*, a 1:8 duty cycle), while discontinued transmission (DTX) was not considered.

#### 2.2.2. Dose Calculation

The total RF-EMF absorbed dose *D* is defined as the sum of the doses due to, on the one hand, the base stations’ downlink signal(s) (*D_DL_*), and on the other, the mobile phone’s uplink signal (*D_UL_*):

(1) $$D=D_{DL}+D_{UL}$$

The downlink dose is calculated as follows:

(2) $$D_{DL}=t_{DL}\times SAR_{DL}\times S_{DL}$$

with *t_DL_* the total time the subject is exposed to the downlink signal (in s), *SAR_DL_* the reference *SAR* (*i.e.*, the *specific absorption* rate; in W/kg per W/m^2^) value for an incident power density of 1 W/m^2^ of a signal with frequency *f*, and *S_DL_* the average power density of the downlink signal (in W/m^2^).

We assumed that during the train ride the subject is continuously exposed to the downlink signal (from either the macrocell or the small-cell base station, or both), and we considered an exposure time *t_DL_* of 1800 s, which is approximately the average duration of a train ride in Belgium. The *SAR_DL_* values were taken from [11], in which a whole-body averaged *SAR_DL_* value of 3.3 mW/kg per W/m^2^ and an organ-specific averaged *SAR_DL_* value for the brain’s gray matter of 3.25 mW/kg per W/m^2^ were obtained for 1800 MHz, by averaging the results of plane waves along six major incident directions (two polarizations). Lastly, the power density *S_DL_* was calculated from the received (downlink) power *P_R_* following ([15], p. 2) where *P_R_* was obtained through calibration of the *RSSI* measurements using the path loss model of Aerts *et al.* [14].

Next, the uplink dose is calculated as follows:

(3) $$D_{UL}=t_{\exp}\times t_{use}\times SAR_{UL}\times P_{UL}$$

with *t_exp_* the total experiment time (in h) (note that, here, *t_exp_* is effectively the same as *t_DL_* in Equation (2)), *t_use_* the use time of the subject’s mobile phone (in s/h), *SAR_UL_* the reference *SAR* value (in W/kg per W) for an uplink power of 1 W (30 dBm) of a signal with frequency *f*, and *P_UL_* the actual uplink power of the signal (in W). In our dose calculations, *t_use_* was varied between 0 and 3600 s/h, and the *SAR_UL_* values were also taken from [11]: for 1,800 MHz a whole-body averaged *SAR_UL_* value of 4.99 mW/kg per W and an organ-specific averaged *SAR_UL_* value of 29.46 mW/kg per W were obtained, by calculating the *SAR* for a human model with an mobile phone placed on the right side of the head.

## 3. Results and Discussion

### 3.1. Power Measurements

#### 3.1.1. Reference Scenario

**Figure 2:**
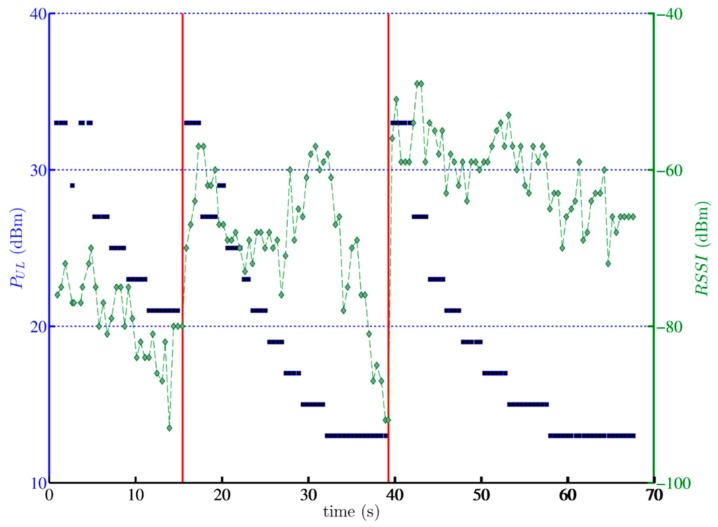
Transmit (*P_UL_*–blue squares) and received power (*RSSI*–green dashed lines with diamonds) during a typical one minute phone call via GSM900 during a train ride. The vertical red lines indicate handovers.

The statistical summary of the measurements in the reference scenario is shown in Table 1. As we were unable to lock the mobile phone to just the GSM1800 band, data was acquired for both GSM900 and GSM1800 connections to the GSM macrocell network (sometimes occurring during the same phone call). The course of a typical call is illustrated in Figure 2 (for GSM900).

**Table 1:** Summary of the received- (*RSSI*) and transmitted- (*P_UL_*) power measurements in the reference scenario, for GSM900 and GSM1800 technologies: number of measurement samples collected per technology (# of samples), and the minimum (min), 5th percentile (p_5_), median, 95th percentile (p_95_) and maximum (max) values.

Band	Power	# of Samples	Min (dBm)	p_5 _(dBm)	Median (dBm)	p_95 _(dBm)	Max (dBm)
GSM900	*RSSI*	30,052	−110	−91	−72	−53	−47
	*P_UL_*	30,052	7	13	21	33	33
GSM1800	*RSSI*	1550	−99	−90	−74	−60	−47
	*P_UL_*	30,052	8	8	18	30	30

At the beginning of the call and immediately after each handover (indicated by red lines in Figure 2), the phone is transmitting at its maximum *P_UL_* of 33 dBm (2 W). A few seconds after these events, *P_UL_* gradually decreases, until, 10 to 20 s later, it more or less stabilizes at 13 dBm (0.02 W). At the same time, *RSSI* follows a more erratic behaviour. The handovers delineate three different cells through which the train passed during the one minute call. In the first cell, *RSSI* fluctuates between roughly −70 dBm and −90 dBm before dropping below approximately −95 dBm just before the handover. This change of connection to a new macrocell base station immediately results in a much higher *RSSI*, fluctuating between approximately −60 and −70 dBm. When the edge of the cell is reached, *RSSI* drops once more, and a new handover occurs. This behavior seems fairly typical, although the *RSSI* values show a lot of variation in any cell. As a side note, the maximum amount of handovers recorded during an established call was five.

For GSM900 (Table 1), the recorded *P_UL_* (7 to 33 dBm) and *RSSI* ranges (−110 to −47 dBm) are very close or equal to the ones reported by Gati *et al.* [16] (5 to 33 dBm, and −110 to −47 dBm, respectively). However, for GSM1800 (Table 1), only the maxima agree ( *RSSI* −47 dBm, and *P_UL_* 30 dBm); the minimum *RSSI* and *P_UL_* found during the train ride are only −99 dBm and 8 dBm, respectively, while minimum values of −110 dBm and 0 dBm were reported in [16]. Additionally, many more samples were obtained for GSM900 (over 20,000 *P_UL_* and 30,000 *RSSI* measurements) than for GSM1800 (about 1,500 measurements of both). Both observations indicate a preference of the mobile phone to connect via the GSM900 band; only when such connection is not available, or the difference in *RSSI* strongly favours GSM1800, the mobile phone connects to the latter. The absence of lower *P_UL_* values (down to 0 dBm) would then be explained due to a lack of time for the mobile phone to further decrease its transmit power before the next handover (back to GSM900) occurs.

Furthermore, the median measured *P_UL_* values of 21 dBm and 18 dBm for GSM900 and GSM1800 (Table 1), respectively, are considerably lower than the median values found in [16] (27 dBm, and 22 dBm). However, in [16], the occurrence of the maximum output power levels was 33% for GSM900 (33 dBm) and 25% for GSM1800 (30 dBm), compared to 13% and 7% in this study. This is probably due to a very high prevalence of short calls in [16]. The median *RSSI* values (Table 1; GSM900: −72 dBm, and GSM1800: −74 dBm), on the other hand, are significantly higher than the ones reported in [16] (GSM900: −88 dBm, and GSM1800: −80 dBm), which seems a surprising result, considering the attenuation effect of the train hull. However, it could be explained by the frequent handovers during the train ride (on average two per minute)–whenever the train leaves the boundaries of the serving cell, the mobile phone switches automatically to the best available connection (*i.e.*, the one with highest *RSSI*).

#### 3.1.2. Small-Cell Scenario

The results of the received-power measurements for the (GSM1800) *small-cell scenario* are listed in Table 2. The transmit power of the mobile phone, *P_UL_*, was constantly at 0 dBm.

**Table 2:** Received-power (*RSSI*) measurements in the small-cell scenario: minimum (min), median, and maximum median values over four orthogonal orientations per position, at distances *d* between 1 and 14 m from the small cell. (The small cell output power *P_SC_* was 17 dBm.)

*d*^1^** (m)	1	2	3	4	6	8	10	12	14
***RSSI*** min (dBm)	−37	−37	−40	−49	−42	−49	−54	−49	−51
***RSSI*** max (dBm)	−30	−28	−29	−25	−33	−35	−36	−40	−41
***RSSI*** median (dBm)	−30	−31	−35	−39	−39	−40	−42	−40	−45

^1^*d* is the distance from the small cell (in m).

As expected, we observed a steady, decreasing trend in median *RSSI* with increasing distance from the small cell within the train, from −30 dBm at 1 m to −45 dBm at 14 m. The median *RSSI* in the car is −40 dBm, which is 34 dB (corresponding to a factor 2500 in received power density) higher than the median and 7 dB (factor 5) higher than the maximum *RSSI* measured in the *reference scenario*.

The mobile phone’s transmit power *P_UL_*, on the other hand, was found to be constant along the corridor, as the mobile phone continually transmitted at its lowest possible output power *P_UL_* of 0 dBm, 18 dB (factor 60) lower than the median and 30 dB (factor 1000) lower than the maximum *P_UL_* recorded in the *reference scenario*.

Whereas *P_UL_* was also invariable across orientations of the experimenter’s body, huge variations in *RSSI* were measured between the orthogonal orientations, due to shadowing of the body, mounting up to 24 dB (factor 250), observed at 4 m from small cell. Moreover, the median *RSSI* along the corridor measured in the frontal orientation was 11 dB (factor 12.5) higher than in the posterior orientation, while both lateral positions showed about 5 dB (factor 3) difference from the former orientation.

Because of the (untunable) high output power of the small cell in our study (17 dBm, or 50 mW) and the consequently high *RSSI* observed in the train, we also calculated the exposure (see Section 3.2) for a scenario involving a small cell with a lower *P_SC_* of 0 dBm (1 mW). Since the *RSSI* in the small-cell scenario scales with *P_SC_*, the median *RSSI* in the train would have been −57 dBm in that case, and the lowest *RSSI* value in the corridor −68 dBm (Table 2). As the latter is still 6 dB higher than the median GSM1800 macrocell coverage (Table 1), the authors assume that sufficient coverage of the whole train car is still guaranteed and 0 dBm would be a realistic value for *P_SC_*. Moreover, because the distance between small cell and mobile phone did not change, the measured *P_UL_* values remain valid.

### 3.2. Exposure Comparison

To compare the exposure of mobile-phone user during the two scenarios (with an additional small-cell scenario featuring a lower *P_SC_*), we calculated the doses absorbed in the whole body and in the brain from the mobile-phone power measurements presented in Section 3.1 (see Table 1 for the reference scenario, and Table 2 for the small-cell scenario). In particular, we used the median *P_UL_* values (correcting for TDMA) and calculated the median power density of the base station signal (*S_DL_*) from the respective *RSSI* measurements (after adding a calibration factor of 3.8 dB to the *RSSI* values to obtain *P_R_*). Furthermore, for illustration, we calculated *D_UL_* absorbed in the whole body and in the brain for a *t_use_* of 9.1 s/h, which was found to be the average call time using 2G devices in the Qualifex study [17]. The results of our dose calculations can be found in Table 3.

**Table 3:** Median downlink power densities (*S_DL_*, in µW/m^2^) and uplink powers (*P_UL_*, in mW) measured in the *reference* and *small cell scenarios* (with small cell output powers *P_SC_* of 0 and 17 dBm) and used in the calculations of the absorbed downlink (*D_DL_*, in mJ/kg) and uplink (*D_UL_*, in mJ/kg) doses for a mobile-phone user on a train ride lasting 30 min (*t_exp_* = 1800 s) and with an average mobile-phone use time *t_use_* of 9.1 s/h. (Results for GSM1800).

			*Whole Body*		*Brain*	
Connection Scenario	*S_DL_* (µW/m^2^)	*P_UL_* ^1^ (mW)	*D_DL_* (mJ/kg)	*D_UL_* (mJ/kg)	*D_DL_* (mJ/kg)	*D_DL_* (mJ/kg)
reference (macro cell network)	0.05	7.89	0.3 × 10^−3^	0.179	0.3 × 10^−3^	1.058
small cell (*P_SC_* = 17 dBm)	110.45	0.13	0.656	0.003	0.646	0.017
small cell (*P_SC_* = 0 dBm)	2.21	0.13	0.013	0.003	0.013	0.017

^1^ TDMA nature of GSM communication (1:8 duty cycle) is taken into account.

**Figure 3:**
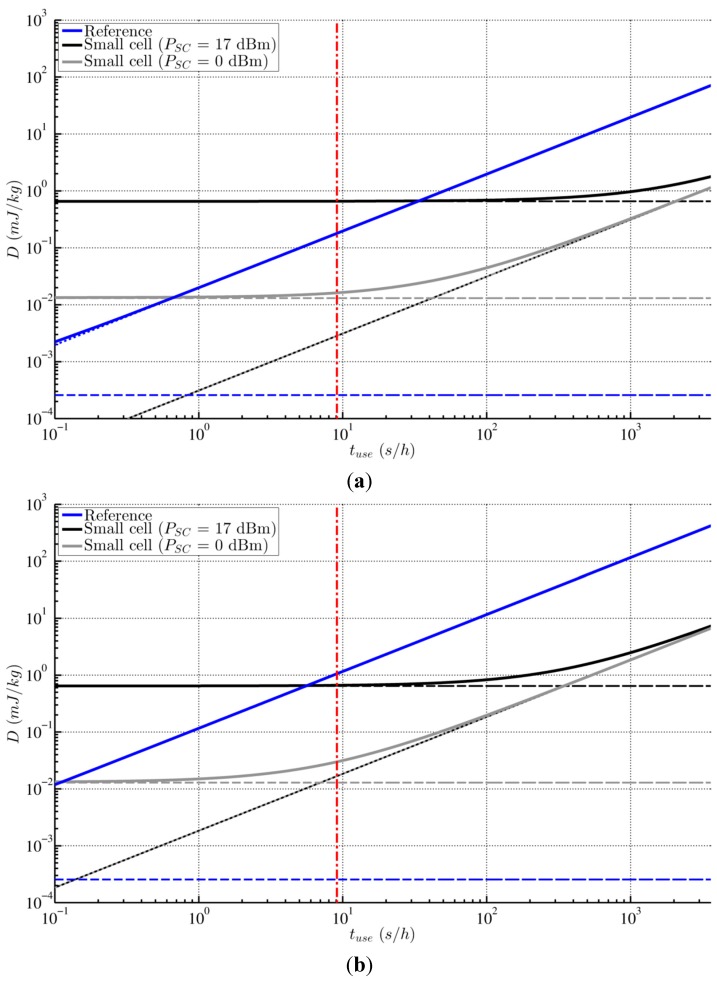
Median RF-EMF doses *D* (full lines), and the respective *D_DL_* (dashed lines) and *D_UL_* (dotted lines), absorbed by a mobile-phone user (**a**) in the body and (**b**) in the brain (gray matter), during a train ride of *t_exp_*= 30 min, as a function of the mobile-phone use time *t_use_*, for the *reference scenario* (macrocell network connection–blue) and for the *small-cell scenario* (small-cell connection, with *P_SC_* either 0 dBm–grey, or 17 dBm–black). The red line represents the 9.1 s/h mark.

Furthermore, we plotted the total absorbed dose *D* (in mJ/kg), as a function of the use time *t_use_* (in s/h), during a 30-minu train ride, in the body (Figure 3a) and in the brain (Figure 3b).

Assuming an average person calls during 9.1 s/h with his mobile phone (see the red line in Figure 3a,b and the values in Table 3), the exposure in the reference scenario due to the subject’s radiating mobile phone is of much larger importance than the downlink exposure (by a factor 600 and 3,500 for the body and the brain, respectively), which is, in fact, negligible (in both scenarios). However, in the small-cell scenario, the results greatly depend on *P_SC_*; in our original small-cell scenario (*P_SC_* = 17 dBm), the resulting downlink doses *D_DL_* are a factor 40 (brain) to 200 (body) higher than the respective *D_UL_* and approximately of the same order as the dominating *uplink* doses in the reference scenario; while for a *P_SC_* of 0 dBm, *D_DL_* are more or less of the same order as *D_UL_*, resulting in total dose that is a factor 11 (body) to 35 (brain) lower than in the reference scenario.

It is clear that the exposure from the base station in the reference scenario (a macrocell) hardly matters, and that the subject’s exposure is *entirely* dependent on *t_use_*. In the small-cell scenario, on the other hand, the magnitude and contribution subdivision of *D* highly depend on the indoor base station’s output power *P_SC_*; the higher *P_SC_*, the higher the contribution of the small-cell downlink exposure, the larger (in terms of *t_use_*) its extent of dominance, and thus the lower the exposure-reducing effect of the small cell. However, for heavy users (e.g., *t_use_* > 1000 s/h, or an 8 min call on a 30 min train ride) *D* becomes nearly independent of *P_SC_* and the exposure reduction reaches its maximum magnitude.

From Figure 3a one can conclude that deploying a small cell in the train would not always result in a reduction of the subject’s total whole-body exposure; more specifically, the exposure will *increase* for high *P_SC_* and low *t_use_*. Alternatively, the brain exposure, as shown in Figure 3b, will almost certainly decrease, e.g., for a use time of at least ~10 s/h for Power Class 1 small cells (maximum *P_SC_* of 20 dBm, or 100 mW) [18].

Additionally, lowering *P_SC_* as much as possible will eventually hit a ceiling in terms of exposure reduction, because *D* will become entirely dependent on *t_use_*. The maximum reduction (in whole-body as well as brain exposure), for GSM1800, in our study is a factor 60.7 (*i.e.*, the ratio of the median *P_UL_* values found in the reference and the small-cell scenario). For illustration (see Table 3), considering the average *t_use_* of 9.1 s/h, the total body exposure in our original small-cell scenario (*P_SC_* of 17 dBm) *increases* by a factor 3.7 (however, the brain exposure is reduced by 150%), while for a *P_SC_* of 0 dBm, the body exposure is reduced by a factor 11 (and the brain exposure by a factor 35).

### 3.3. Strengths and Limitations

The framework applied in this study can be used to add the contributions of far-field and near-field RF sources to the total RF-EMF exposure (in the body or in any organ) of an individual, thus enabling us to compare multiple scenarios involving any number and types of base stations, users, user devices, and time frames in order to minimize the subject’s total exposure. However, the considered framework is not meant to compare the subject’s exposure to the guidelines issued by international organizations such as ICNIRP [19] or the legal limits in regions or countries.

In comparison with the results of GSM1800 downlink measurements in the train found in literature, we obtained a much lower *S_DL_* in our reference scenario (0.05 µW/m^2^); e.g., Bolte and Eikelboom [6] measured an average power density of 7 µW/m^2^. This could be explained by (i) the fact that we measured the contribution of only one carrier frequency; (ii) the measurement device used in [6], which has a sensitivity of only 6.7 µW/m^2^; or (iii) our conversion from *RSSI* to *S_DL_*, or by a combination of these factors. However, even when considering this *S_DL_* of 7 µW/m^2^ in our reference scenario (and thus a *D_DL_* of ~0.03 mJ/kg), the uplink radiation still accounts for most of the user’s exposure (for *t_use_* 9.1 s/h: 86% in the body, 97% in the brain), and the heavy dependence of the total dose *D* on *t_use_* remains true–although less pronounced for the whole-body exposure and for very-light mobile phone users (*t_use_* < 10 s/h, or a 5 s call during a 30 min train ride). Moreover, although the maximum magnitude of the exposure reduction in the small-cell scenario (factor 60.7) does not change, the small cell will commence to have a beneficiary effect on the exposure for lower *t_use_* and/or higher *P_SC_*.

It’s important to keep in mind that the contribution of the mobile phones of *other* people in the train to the far-field exposure of a person (in this study downlink and far-field exposure are interchangeable, but with the addition of other users, their uplink signals add to the subject’s far-field exposure) can be quite significant. In fact, in the study by Bolte and Eikelboom [6], this contribution was found to be more than 10 times higher (92 µW/m^2^) than the base stations’ contribution (7 µW/m^2^), and Plets *et al.* [20] calculated that in a train car with 15 (average) users, their uplink signals add to the (average) subject’s total exposure up to 24% of his own uplink exposure. Taking into account other users’ mobile phones, we thus find that the small cell has an increased beneficiary effect on the exposure of passengers who are light users (whose exposure is dominated by the contributions of the small cell base station and/or other passengers’ mobile phones), and a comparable effect on the exposure of heavy users (whose exposure is dominated by their own mobile phone) (with the maximum reduction unaffected), compared to a scenario without other users. Additionally, it is probable that the small cell radiates more power when more users are connected to it, as is the case with macrocell base stations. However, assuming the *P_SC_* values above are average values over the train ride, we do not have to keep account for this in our analysis.

Furthermore, as additional measurements with a dosimeter inside the train during this study did not reveal a relevant UMTS downlink contribution, we did not consider the small-cell-to-macrocell-network connection antenna on the roof of the train as an RF source in our exposure calculations. This is probably due to its antenna pattern and the shielding of the train roof.

In our dose calculations, we disregarded GSM’s DTX mode, which becomes active when no voice is transmitted, and in which the mobile phone saves battery power by entering a 1:69 duty cycle (instead of its regular 1:8 TDMA structure). As a result, the actual uplink dose can be lower in practice (by up to a factor 8.5) moving the point at which the small cell becomes beneficiary to the user’s exposure to slightly higher *t_use_* and/or lower *P_SC_*.

We are confident that using two different mobile phones for our measurements does not significantly bias our exposure comparison. In this study, we attempted at a general comparison of the exposure in a macrocell and a small-cell scenario for an average GSM1800 mobile-phone user on the train. In practice, there are many different types of mobile phones, and it is impossible to account for all small differences in their effective output power or the absorption of their radiation in the body and the brain.

It should further be noted that all doses were averaged over sizeable volumes: the whole body or the brain’s grey mass. For the downlink dose, this is merely logical, as the incident fields impact the body (relatively) evenly. The uplink signal, on the other hand, will mostly be absorbed locally, in a much smaller volume (e.g., in the head when calling). However, this averaging over a larger volume is necessary to compare and add the two contributions to the total exposure. In any case, all conclusions concerning the uplink dose *D_UL_* in the brain will also be applicable to the localized dose (though even more pronounced).

## 4. Conclusions

The influence of a small cell on a mobile-phone user in a train is twofold. On the one hand, its vicinity to the passengers could result in a substantially increased downlink exposure; however, this is highly dependent on its effective output power. On the other hand, for the same reason, and also due to the elimination of handovers, the transmit power of any mobile phone will be considerably lower, significantly reducing the exposure due to one’s mobile device (and those of others). Combining both exposure factors, it is found that, in a realistic one-user scenario for GSM1800, the user’s total exposure of the body can be reduced by a factor 11, and of the brain by a factor 35; while both could be maximally reduced by a factor 60.

However, whether the total human RF-EMF exposure in the train due to mobile communications is reduced by the deployment of a small cell ultimately depends on several factors, including the output power of the small cell, the number of small cells in the train (depending on how many simultaneous users have to be served), as well as the number of users in the train, and how long they use their devices. This will be the subject of future research.
